# Kidney Transplantation After Rituximab Treatment for End-Stage Renal Failure With Myeloperoxidase Anti-neutrophil Cytoplasmic and Anti-glomerular Basement Membrane Antibody Positivity: A Case Report

**DOI:** 10.7759/cureus.94237

**Published:** 2025-10-09

**Authors:** Takuya Sugiura, Akihito Tanaka, Nobuhiro Nishibori, Takaya Ozeki, Yuka Sato, Kayaho Maeda, Kazuhiro Furuhashi, Noritoshi Kato, Tomoki Kosugi, Yuta Sano, Shohei Ishida, Shoichi Maruyama

**Affiliations:** 1 Department of Nephrology, Nagoya University Graduate School of Medicine, Nagoya, JPN; 2 Department of Nephrology, Nagoya University Hospital, Nagoya, JPN; 3 Department of Urology, Nagoya University Graduate School of Medicine, Nagoya, JPN

**Keywords:** anti-glomerular basement membrane antibody, double-positive, kidney transplant, myeloperoxidase anti-neutrophil cytoplasmic antibody, renal failure

## Abstract

Double positivity for myeloperoxidase anti-neutrophil cytoplasmic antibody (MPO-ANCA) and anti-glomerular basement membrane (anti-GBM) antibody is associated with distinct clinical features, including older age at onset, prolonged symptom duration, and a combination of severe renal involvement and pulmonary hemorrhage. Although kidney transplantation in patients with isolated anti-GBM disease carries a low risk of recurrence, the risk in double-positive patients remains uncertain, warranting careful immunosuppressive management and close monitoring. Herein, we report the case of a 28-year-old female patient who developed end-stage kidney disease due to rapidly progressive glomerulonephritis with MPO-ANCA and anti-GBM double positivity. Following hemodialysis and infection-related complications, treatment with rituximab was initiated to suppress the progression of MPO-ANCA-associated vasculitis. Subsequently, she underwent ABO-compatible kidney transplantation, with her father as the donor. One year after transplantation, graft function remained stable, without evidence of recurrence or rejection on protocol biopsy. This case highlights the safety of kidney transplantation in double-positive patients with appropriate immunosuppressive strategies, including pretransplant rituximab, even when performed relatively early after diagnosis. Our experience suggests the potential for favorable outcomes in this high-risk subgroup, although further data are needed to establish optimal timing and treatment approaches.

## Introduction

Patients who are double-positive for myeloperoxidase anti-neutrophil cytoplasmic antibody (MPO-ANCA) and anti-glomerular basement membrane (anti-GBM) antibody exhibit distinct characteristics from those with single antibody positivity [1,2]. Double-positive patients tend to be older at disease onset and experience a longer duration of symptoms before diagnosis than patients with anti-GBM antibody positivity alone [3]. Clinically, these patients present with a combination of features characteristic of ANCA-associated vasculitis (AAV), such as older age and prolonged symptom duration, along with manifestations typical of anti-GBM disease, including severe renal impairment and a high incidence of pulmonary hemorrhage [4]. Double-positive patients have been reported to have a high rate of nephritis recurrence [3].

Kidney transplantation in patients with double positivity for MPO-ANCA and anti-GBM antibodies is rare, and data on recurrence risk remain limited. Although the post-transplant recurrence rate in patients with anti-GBM disease alone is low [5], double-positive patients are thought to be at risk of AAV relapse, necessitating continued immunosuppressive therapy and careful monitoring after transplantation [3].

Here, we report the case of a young female with double positivity for MPO-ANCA and anti-GBM antibodies who developed end-stage renal disease due to rapidly progressive glomerulonephritis. The patient successfully underwent kidney transplantation following rituximab therapy with no evidence of disease recurrence. We present this case to highlight the feasibility and safety of kidney transplantation in a double-positive MPO-ANCA and anti-GBM patient following rituximab therapy, with no recurrence observed during follow-up.

## Case presentation

A 28-year-old female patient with renal dysfunction was admitted for emergency care. At the age of 26, she was diagnosed with depression and regularly attended the psychiatry department in the same hospital. Her depression was well controlled with sertraline at a dose of 25 mg/day. Approximately six months before admission, she visited the clinic with a cold, at which time her serum creatinine was 0.6 mg/dL. Approximately one month before admission, she experienced rapid weight gain of 5 kg over several days.

During routine psychiatric follow-up, on the day of admission, the patient reported leg edema. The psychiatrist therefore performed urinalysis and blood tests, which revealed renal dysfunction. Laboratory tests showed a serum creatinine of 8.65 mg/dL, a urinary protein-to-creatinine ratio of 4.1 g/g creatinine, > 50 red blood cells per high-power field in the urine, an MPO-ANCA level of 19.3 U/mL, and an anti-GBM antibody level of 2.9 U/mL (Table 1). She was thus admitted to the hospital on the same day for emergency care.

**Table 1:** Laboratory findings on admission Reference ranges are representative values based on commonly used clinical laboratory standards and may vary slightly depending on the laboratory and assay method. HDL: high-density lipoprotein; LDL: low-density lipoprotein; CH50: 50% hemolytic complement (total complement test); C3: omplement component 3; C4: omplement component 4; IgG: immunoglobulin G; IgA: immunoglobulin A; IgM: immunoglobulin G; ANCA: antineutrophil cytoplasmic antibodies; GBM: glomerular basement membrane

Parameter	Patient value	Reference range	Interpretation
White blood cell count (/µL)	6,000	3,500–9,000	
Neutrophils (%)	70.1	40–70	High
Lymphocytes (%)	19.6	20–45	Low
Monocytes (%)	6.9	2–8	
Eosinophils (%)	3.2	1–6	
Basophils (%)	0.2	0–1	
Red blood cell count (×10⁴/µL)	310	420–560	Low
Hemoglobin (g/dL)	8.7	13.0–16.5	Low
Hematocrit (%)	27.3	39–52	Low
Mean corpuscular volume (fL)	88.1	80–100	
Mean corpuscular hemoglobin (pg)	28.1	27–33	
Mean corpuscular hemoglobin concentration (g/dL)	31.9	32–36	Low
Reticulocyte (‰)	8.6	2–12	
Platelet count (×10⁴/µL)	15.1	15–35	
Total protein (g/dL)	5.8	6.5–8.0	Low
Albumin (g/dL)	2.9	3.8–5.0	Low
Total cholesterol (mg/dL)	224	120–220	High
Glucose (mg/dL)	106	70–109	
Blood urea nitrogen (mg/dL)	80.7	8–20	High
Serum creatinine (mg/dL)	8.65	0.6–1.2	High
Estimated GFR (mL/min/1.73m²)	5.2	>60	Low
Total bilirubin (mg/dL)	0.4	0.2–1.2	
Amylase (U/L)	55	40–120	
Creatine kinase (U/L)	122	60–220	
HDL cholesterol (mg/dL)	53	40–90	
LDL cholesterol (mg/dL)	155	<140	High
Hemoglobin A1c (%)	5.5	4.6–6.2	
Lactate dehydrogenase (U/L)	210	120–245	
C-reactive protein (mg/dL)	0.49	<0.3	High
Sodium (mEq/L)	136	135–145	
Potassium (mEq/L)	6.1	3.5–5.0	High
Chloride (mEq/L)	105	98–108	
Calcium (mg/dL)	8.0	8.6–10.2	Low
Phosphate (mg/dL)	7.3	2.5–4.5	High
Magnesium (mg/dL)	2.5	1.8–2.5	
Bicarbonate (mmol/L)	18.9	22–29	Low
CH50 (U/mL)	57.5	30–45	High
C3 (mg/dL)	94.9	86–160	
C4 (mg/dL)	29.7	17–45	
IgG (mg/dL)	929	870–1700	
IgA (mg/dL)	211	110–410	
IgM (mg/dL)	124	35–220	
Antinuclear antibody	<40	<40	
Myeloperoxidase-ANCA (U/mL)	19.3	<3.5	High
Proteinase 3-ANCA (U/mL)	<1.0	<3.5	
Anti-GBM antibody (U/mL)	2.9	<1.0	High
Erythropoietin (mIU/mL)	5.7	4.2–27.8	
Thyroid-stimulating hormone (μIU/mL)	3.77	0.5–5.0	
Free triiodothyronine (pg/mL)	1.87	2.3–4.0	Low
Free thyroxine (ng/dL)	0.99	0.9–1.7	
Intact parathyroid hormone (pg/mL)	242	10–65	High
Hepatitis B surface antibody	Negative	Negative	
Hepatitis B core antibody	Negative	Negative	
Hepatitis C virus antibody	Negative	Negative	
Specific gravity	1.012	1.005–1.030	
pH	5.5	5.0–7.5	
Protein	4+	Negative	High
Urine protein-to-creatinine ratio (g/gCre)	5.41	<0.15	High
Glucose	Negative	Negative	
Occult blood	3+	Negative	High
Ketone	Negative	Negative	
White blood cell elastase	2+	Negative	High
Nitrate	Negative	Negative	
β₂-microglobulin (μg/L)	34,877	<230	High
α₁-microglobulin (mg/L)	41	<10	High
N-acetyl-β-D-glucosaminidase (IU/L)	15.1	<7.0	High
Red blood cells (/HPF)	>50	<5	High
White blood cells (/HPF)	5–9	<5	High

A renal biopsy was performed on the second day of hospitalization. There were 20 visible glomeruli, eight of which showed global sclerosis. One fibrocellular crescent, one fibrous crescent, and 10 cellular crescents were observed; most were circumferential with severely collapsed capillary loops. Endocapillary hypercellularity was observed in some areas; however, no adhesions were observed. Interstitial fibrosis and tubular atrophy (IFTA) accounted for > 90% of the cortical interstitium. Inflammatory cell infiltration, mainly of mononuclear cells, was observed in areas of the IFTA and around the glomeruli with cellular crescents. No intimal thickening or hyalinosis was observed in any arteries. Immunofluorescence staining revealed linear deposition of immunoglobulin (Ig) G and C3 along the GBM. The IgG subclasses were deposited in the following order: IgG1, IgG2, IgG3, and IgG4. κ and λ light chains were also positive in parts of the tubular basement membrane and within the tubular lumens. No electron-dense deposits were observed. Based on these findings, the cause of acute kidney injury was diagnosed as anti-GBM antibody disease.

Given the irreversible nature of her renal dysfunction, hemodialysis was initiated, and ABO-compatible kidney transplantation was planned with her father as the donor. Her 61-year-old father was blood group-compatible, and donor-specific antibodies were negative. The patient tested positive for anti-hepatitis B core (HBc) antibodies and negative for anti-hepatitis B surface antibody (HBs) and hepatitis B (HB) virus DNA, indicating an inactive HB carrier status. No other contraindications for donation were identified.

A human leukocyte antigen (HLA) typing test revealed three mismatches, with one each at loci A, B, and DR. Complement-dependent cytotoxicity (CDC) showed that T warm was negative, B warm was negative, and B cold was positive. Flow cytometry cross-matching (FCXM) measured T cells at 0.95 (ratio of sample median to negative control median value, cut-off < 2.0) and B cells were 3.07 (ratio of sample median to negative control median value, cut-off < 2.0). Screening for anti-HLA antibodies (LABScreen Mixed; Thermo Fisher Scientific, Inc., Waltham, Massachusetts, United States) showed class 1:0.9 (normalized background ratio, cutoff < 1.5) and class 2:1.0 (normalized background ratio, cutoff < 1.5). She had no history of sensitization, such as blood transfusion or pregnancy. Considering these findings, the results of CDC for B cold and FCXM for B cells were considered non-specific reactions.

On the third hospital day, a temporary dialysis catheter was inserted, and hemodialysis was initiated. On day 43, a tunneled dialysis catheter was placed, and the patient was discharged on day 52. However, she was readmitted on day 67 with methicillin-sensitive *Staphylococcus aureus* bacteremia due to a catheter-related infection. After replacing the catheter and completing antibiotic therapy, she was discharged on day 99 but required another hospitalization on day 116 for recurrent catheter infection. The catheter was removed, and her condition improved with the administration of antibiotics.

**Figure 1:**
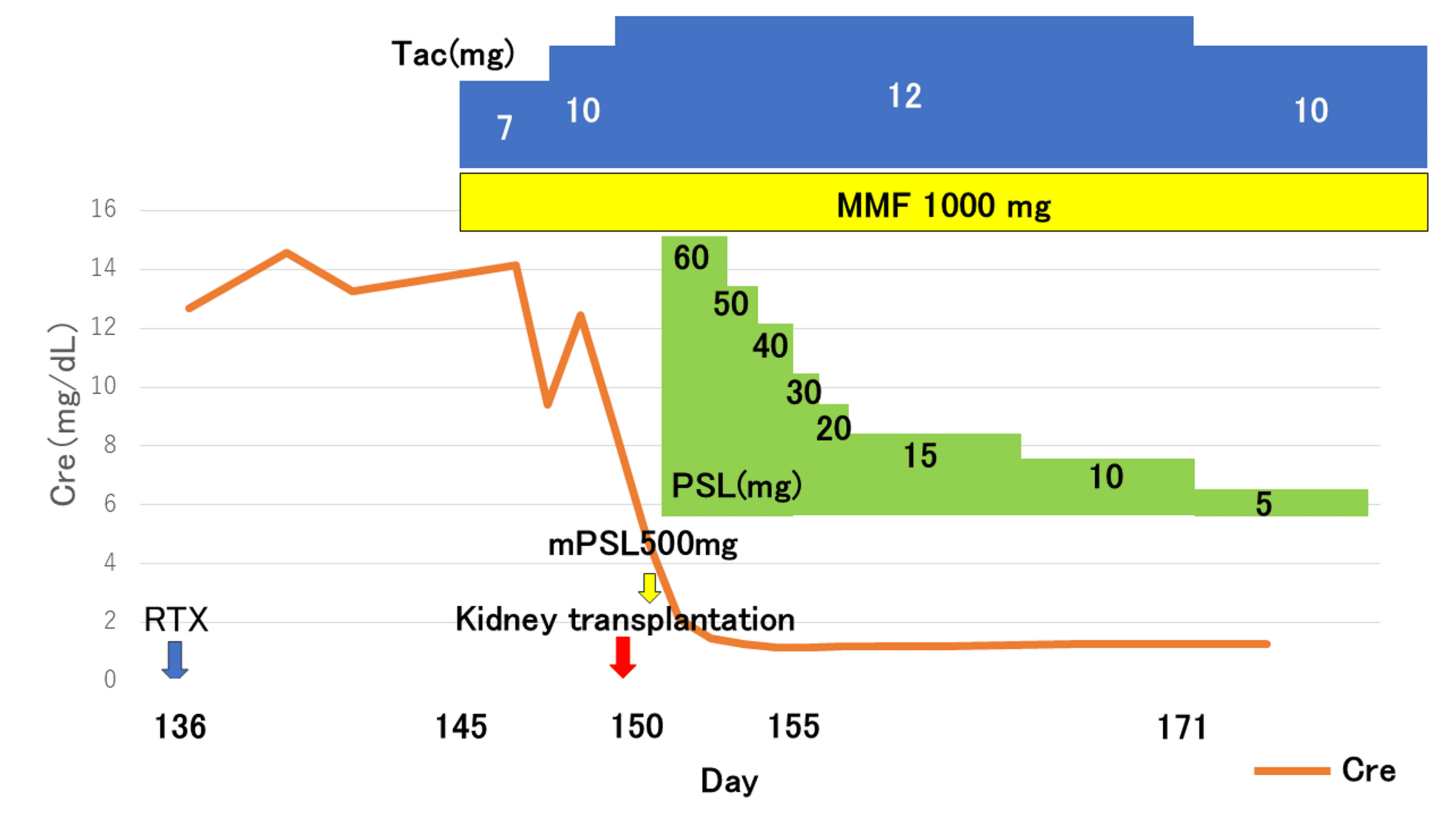
Clinical course of the patient The figure illustrates the immunosuppressive medications administered before and after kidney transplantation, along with the corresponding changes in renal function, as indicated by serum creatinine levels. Following transplantation, serum creatinine levels rapidly decreased and remained stable thereafter. Cre, creatinine; mPSL, methylprednisolone; PSL, prednisolone; Tac, tacrolimus; MMF, mycophenolate mofetil; RTX, rituximab

Owing to these catheter-related complications and prolonged hospitalization, we determined that early kidney transplantation was desirable. On day 150 of hospitalization, she received rituximab (500 mg), followed by tacrolimus, mycophenolate mofetil, and methylprednisolone. Rituximab was prescribed because of concerns that MPO-ANCA could potentially drive the production of anti-GBM antibodies. Kidney transplantation was performed on day 165. Her preoperative serum creatinine was approximately 14.0 mg/dL; postoperative urine output was promptly established, and her creatinine level improved to approximately 1.3 mg/dL (Figure 1).

Protocol kidney biopsy at one year post transplant indicated no evidence of recurrent glomerulonephritis or rejection, and the patient remained stable. 

## Discussion

The recurrence of anti-GBM antibody disease after kidney transplantation has been reported infrequently [6,7]. However, cases such as this, in which MPO-ANCA and anti-GBM antibodies are simultaneously positive, have been suggested to have a higher recurrence rate of glomerulonephritis and worse renal prognosis than cases in which only anti-GBM antibodies are positive [3,8]. Although reports of kidney transplantation in double-positive cases are limited, the risk of recurrence may be higher than that in cases with anti-GBM antibody positivity alone.

Regarding the mechanism underlying the coexistence of these two autoantibodies, it is hypothesized that preexisting ANCA, particularly MPO-ANCA, activates neutrophils, leading to inflammatory injury to the glomerular capillary walls [9]. This injury exposes structural proteins of the GBM, especially the NC1 domain of α3(IV) collagen, which is normally sequestered from the immune system. As a result, an immune response against these epitopes is triggered, leading to the production of anti-GBM antibodies [10-12].

In the present case, anti-GBM antibody test results were nearly negative at the time of diagnosis. Because the disease was confined to the kidneys and renal damage was irreversible, treatment with steroids, immunosuppressive agents, or plasma exchange was not performed, and renal replacement therapy with hemodialysis was initiated. Considering that MPO-ANCA could potentially drive the production of anti-GBM antibodies, rituximab was administered before transplantation to suppress antibody production.

It is generally recommended that kidney transplantation should be performed for anti-GBM antibody disease only after confirming that the disease has been in remission for at least six months and that the anti-GBM antibody has remained negative for over 12 months [13]. In this case, although a longer waiting period might have further reduced the risk of recurrence, prolonged hospitalization due to catheter-related complications prompted us to proceed with transplantation slightly earlier, approximately 150 days after diagnosis. The patient remained recurrence-free, suggesting that even in double-positive cases, kidney transplantation can be safely performed under appropriate immunosuppressive management.

## Conclusions

Our case suggests that kidney transplantation can be performed safely in patients double-positive for MPO-ANCA and anti-GBM antibodies when pretransplant rituximab and individualized immunosuppressive strategies are applied. Although the evidence base remains limited, this report contributes to the growing experience indicating that favorable outcomes may be achievable in this uncommon clinical context.
